# Temporary Diverting Stoma Improves Recovery of Anastomotic Leakage after Anterior Resection for Rectal Cancer

**DOI:** 10.1038/s41598-017-16311-7

**Published:** 2017-11-21

**Authors:** Yuchen Wu, Hongtu Zheng, Tianan Guo, Adili Keranmu, Fangqi Liu, Ye Xu

**Affiliations:** 1 Department of Colorectal Surgery, Fudan University Shanghai Cancer Center, No. 270, Dong An Road, Shanghai, 200032 China; 2 Department of Oncology, Shanghai Medical College, Fudan University, No. 130, Dong An Road, Shanghai, 200032 China

## Abstract

Temporary diverting stoma might be a protective factor for the prevention of anastomotic leakage (AL) after anterior resection. Its role in leakage recovery is unknown. This study aimed to evaluate the effect of temporary diverting stoma on anastomotic leakage severity and recovery. We analyzed 323 patients who underwent anterior resection for rectal cancer and developed anastomotic leakage, in which 44 had temporary diverting stoma. Association between diverting stoma and occurrence of anastomotic leakage, recovery time, length of hospital stay, overall costs, local and distant relapse-free survival were further studied. In non-severe AL group, temporary diverting stoma improved leakage recovery by 4 days (mean: 20.7 days vs. 16.1 days, *p* = 0.031), especially in patients who did not receive neoadjuvant treatment (mean time: 20.9 days vs. 14.4 days, *p* = 0.016). However, it did not delay the occurrence of anastomotic leakage. Moreover, no significant difference was found in the overall length of hospital stay and costs among patients with versus without a diverting stoma. In severe AL group, however, no difference was detected. The advantage of shortened leakage recovery did not reduce the local and distant relapse-free survival. In conclusion, our findings indicated the recovery benefit from diverting stoma in patients with anterior resection.

## Introduction

Anastomotic leakage (AL) is a prevalent complication after anterior resection of rectal tumors, occurring in 5% to 19% of patients according to various institutional reports^1^. It may incur adverse morbidity and mortality postoperatively^2,3^ as well increased economic burden^4^. Moreover, ALhas been confirmed to be associated with increased local recurrence^5–7^.

Many pre- and post-surgical factors are associated with increased risk of AL, including male sex, The American Society of Anesthesiologists (ASA)score >2, history of neoadjuvant chemoradiotherapy (neo-CRT), and intraoperative blood transfusion^8,9^.On the contrary, temporary diverting stoma, including ileostomy and transverse colostomy, is associated with a decreased incidence of AL. A systematic review of 11 studies showed a reduced rate of AL in low anterior resections(relative risk: 0.38, 95% confidence interval (CI):0.30–0.48, *p* < 0.001) when a diverting stoma was applied^9^.

Most studies thus far have investigated the etiology of AL, as well as the controversy about whether temporary could reduce the risk of leakage. However, few have focused on its impact on recovery process and prognosis after AL occurs. The aim of this study was to evaluate diverting stoma as a factor affecting AL recovery in patients after anterior resection of rectal tumors.

## Results

### Patient characteristics

The incidence of AL in our study was 6.2%. Table 1 summarizes patients’ clinicopathologic features. Patients were predominantly male (81.9%), and the mean age was 58.0 years. Overall, 26.2% and 15.9% of patients had a history of excess smoking and alcohol intake, respectively. Median BMI was 23.8. Forty three patients were treated with neo-CRT before the primary operation. During the operation, only 2 patients required blood transfusion. Distance from the anastomosis to the anal verge did not exceeded 5 centimeters in 45 (13.9%) patients. Forty four patients had temporary diverting stoma.

**Table 1:** Patient Characteristics.

**Patient Characteristics (N = 323)**	**N (%)**
Age, mean ± SD,yr	58.0 ± 11.2
Sex: Male	265 (81.9)
**Personal History**	
Smoking (>40 pack-years)	85 (26.2)
Long-Term Alcohol History	51 (15.9)
BMI, mean ± SD, kg/m^2^	23.8 ± 3.4
**Systems Review**	
Vascular disease	77 (23.9)
Diabetes	48 (14.9)
ASA Score^a^ (1–2)	162 (50.2)
Weight Lost ≥5 kg	37 (11.5)
Anemia	40 (12.3)
Neo-CRT^b^	43 (13.3)
**Operation**	
Type of Surgery: Urgent	20 (6.1)
Blood Transfusion	2 (0.6)
Temporary Diverting Stoma	44 (13.6)
Distance: Low Rectum (≤5 cm)	278 (86.1)
**Pathology after Operation**	
TNM Stage	
0	6 (2.0)
1	68 (21.0)
2	85 (26.3)
3	145 (44.8)
4	18 (5.9)
Lymphovascular Invasion	65 (20.1)
Perineural Invasion	58 (18.0)
Extranodal Tumor Deposits	45 (13.9)
Primary Maximum Diameter, mean ± SD, cm	4.1 ± 1.9
**Leakage Type**	
Severe	38 (11.8)
Non-Severe	285 (88.2)

^a^American Society of Anesthesiologists Score; ^b^Neoadjuvant Chemoradiotherapy.

### Association of variables with temporary diverting stoma

The application of temporary diverting stoma during anterior resection was based on surgeons’ experience and their evaluation of the patients’ condition. In order to investigate the possible reasons for the decision regarding use of a diverting stoma, we compared the association of diverting stoma with other variables (Table 2). Treatment with neo-CRT solely correlated with the use of a diverting stoma (*p* = 0.002). Other factors did not correlate with the use of a diverting stoma.

**Table 2:** Association of Variables in Patients with or without Temporary Diverting Stoma.

**Association of Variables in Patients with or without Temporary Diverting Stoma (N = 323)**			
**Variables**	**Patient with (N = 44) (%)**	**Patients without (N = 279) (%)**	***P***
Age, mean ± SD, yr	59.3 ± 10.9	58.1 ± 11.3	NS
Sex: Male	36 (81.8)	229 (82.1)	NS
Smoking (>40 pack-years, Yes)	15 (34.1)	70 (25.1)	NS
Long-Term Alcohol History (Yes)	10 (22.7)	41 (14.7)	NS
BMI, mean ± SD, kg/m^2^	23.7 ± 3.2	23.8 ± 3.4	NS
Vascular disease (Yes)	13 (29.5)	64 (22.9)	NS
Diabetes (Yes)	8 (18.2)	40 (14.3)	NS
ASA Score (1–2)	24 (54.5)	138 (49.5)	NS
Weight Lost ≥5 kg (Yes)	8 (18.2)	38 (13.6)	NS
Anemia (Yes)	7 (15.9)	30 (10.8)	NS
Neo-CRT (Yes)	13 (29.5)	30 (10.8)	**0.002**
Type of Surgery: Urgent (Yes)	3 (6.8)	17 (6.1)	NS
Blood Transfusion (Yes)	1 (2.3)	1 (0.3)	NS
Distance: ≤5 cm	39 (88.6)	239 (85.7)	NS
Primary Maximum Diameter, mean ± SD, cm	4.2 ± 1.2	4.0 ± 1.5	NS

A two-tailed p value ≤ 0.05 was considered statistically significant; NS, not significant.

### Diverting stoma effect on the recovery of non-severe AL

Considering that various therapy strategies were applied to patients with severe or non-severe AL, we discussed the recovery of AL separately in these two groups.

In non-severe groups, male patients were more likely to develop early AL than female patients (Supplementary Table S1, mean: 6.6 days vs. 7.5 days, *p* = 0.042). However, occurrence time did not differ in patients with or without temporary diverting stoma.

We have found that only neo-CRT correlated with the presence of a diverting stoma. To remove the possible influence and bias of neo-CRT on the presence of a diverting stoma, a grouped analysis was conducted (Table 3). Occurrence time did not differ among all 285 patients (mean: 6.5 days vs. 7.0 days, *p* = 0.302) who did or did not receiveneo-CRT. However, a shorter recovery time was detected (mean: 16.1 days vs. 20.7 days, *p* = 0.031), especially in patients who did not receive neo-CRT (mean: 14.4 days vs. 20.9 days, *p* = 0.016). No significant difference was detected in the neo-CRT group. Moreover, no difference was found among other variables (Supplementary Table S1).

**Table 3:** Time Evaluation of AL in Non-Severe Group.

**Time Evaluation of AL in conservative therapy after Temporary Diverting Stoma (N = 285/323)**									
**Diverting Stoma**	**All Patients**			**Patients without Neo-CRT**			**Patients with Neo-CRT**		
	**With (N = 40)**	**Without (N = 245)**	***P***	**With (N = 24)**	**Without (N = 221)**	***P***	**With (N = 16)**	**Without (N = 24)**	***P***
Occurrence Time, (mean ± SD, d)	6.5 ± 2.3	7.0 ± 3.2	NS	6.7 ± 2.6	6.9 ± 2.4	NS	5.7 ± 1.8	8.0 ± 5.2	NS
LOH, (mean ± SD, d)	16.1 ± 8.6	20.7 ± 12.3	**0.031**	14.4 ± 7.9	20.9 ± 12.5	**0.016**	19.3 ± 9.9	19.2 ± 10.9	NS

A two-tailed p value ≤ 0.05 was considered statistically significant; NS, not significant.

### Length of hospital stay and economic benefit in non-severe group

Among all patients with conservative treatment, the mean length of hospital stay of patients with a diverting stoma was 22.1 days, including secondary hospitalization for reoperation of stoma closure, while that of patients without a diverting stoma was 21.7 days. No difference was detected (Table 4). Overall cost for hospital treatment did not differ between groups.

**Table 4:** LOH and Cost in Non-Severe Group.

Length of Hospital Stay and Cost in Patients of with or without Diverting Stoma (N = 285)			
Variables	Patients with (N = 40)	Patients without (N = 245)	*P*
LOH, mean ± SD, d	22.1 ± 12.8	21.7 ± 12.0	NS
Cost, mean ± SD, $	8897.7 ± 4819.4	9429.7 ± 3168.7	NS

A two-tailed p value ≤ 0.05 was considered statistically significant; NS, not significant.

### Impact of diverting stoma on severe AL and time evaluation

Temporary diverting stoma did not decrease cases in severe group, compared to all patients with AL. (Table 5) Moreover, mean occurrence time of severe AL ranged from 3.3 to 5.3 days, while mean LOH from 22.3 to 24.7 days. No significant difference in occurrence time and LOH, whether in patients without Neo-CRT, was detected (Table 6). We did not investigated them in patients with Neo-CRT because only 1 patients left in this group.

**Table 5:** Impact of Diverting Stoma on Incidence of Severe AL.

Incidence of Severe AL (N = 38/323)						
	All Patients			Patients without Neo-CRT		
Diverting Stoma	With (N = 4)	Without (N = 34)	P	With (N = 3)	Without (N = 32)	P
Incidence	4/44 (9.1%)	34/278 (12.2%)	NS	3/27 (11.1%)	32/253 (12.6%)	NS

A two-tailed p value ≤ 0.05 was considered statistically significant; NS, not significant.

**Table 6:** Time Evaluation of AL in Severe Group.

Time Evaluation of AL in severe AL after Temporary Diverting (N = 38)						
Diverting Stoma	All Patients			Patients without Neo-CRT		
	With (N = 4)	Without (N = 34)	P	With (N = 3)	Without (N = 32)	P
Occurrence Time, mean ± SD, d	3.3 ± 4.0	5.3 ± 2.6	NS	4.3 ± 4.1	5.3 ± 2.7	NS
LOH, mean ± SD, d	24.7 ± 7.4	23.1 ± 12.1	NS	24.7 ± 7.4	22.3 ± 11.6	NS

A two-tailed p value ≤ 0.05 was considered statistically significant; NS, not significant.

### Survival analysis

**Figure 1:**
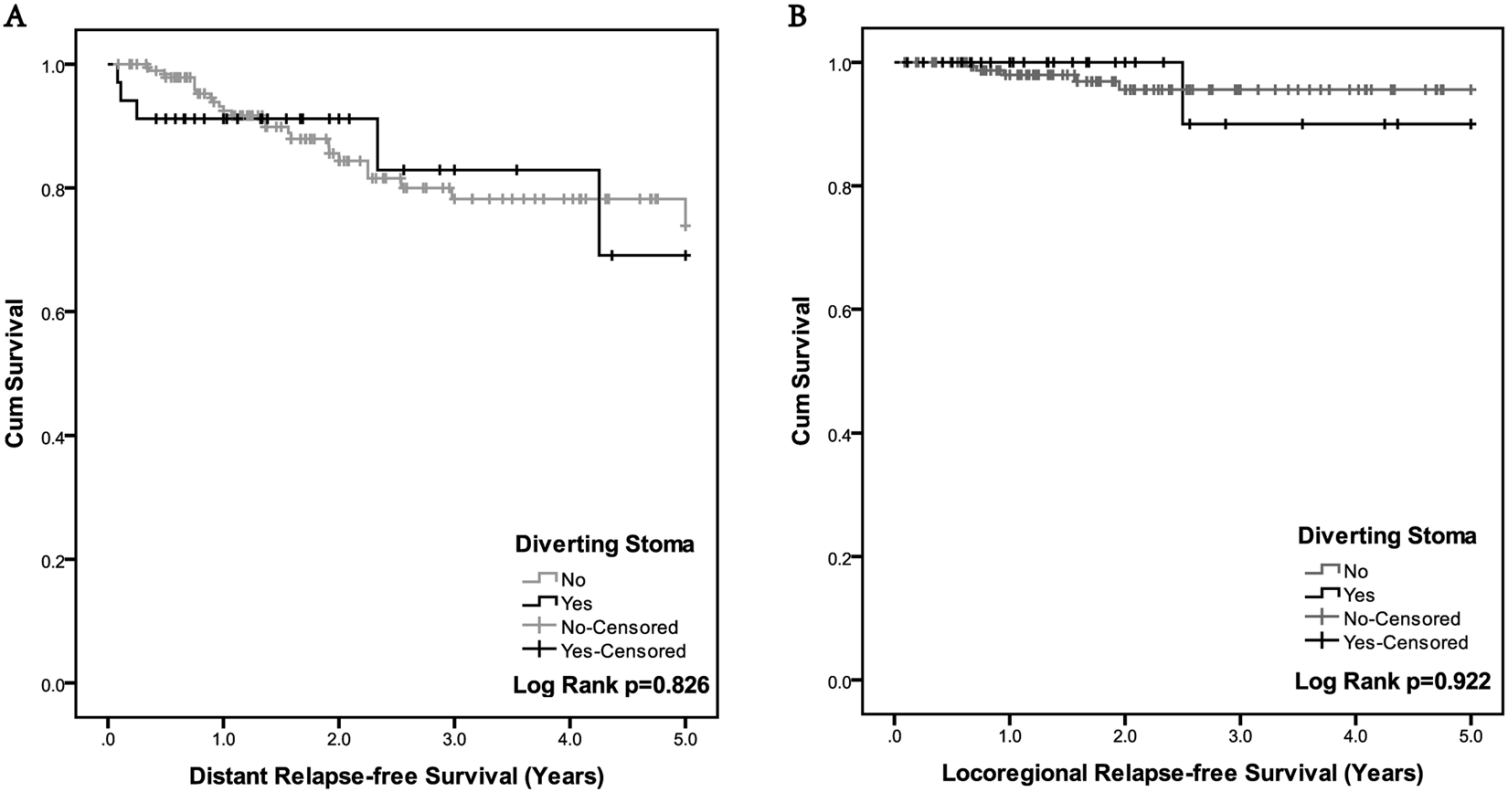
Survival curve of patients with (N = 34) or without (N = 196) diverting stoma. No significant difference was found of both distant relapse-free survival (1 A, p = 0.826) and locoregional relapse-free survival (1B, p = 0.922) in patients with or without temporary diverting stoma.

Data from 230 patients was analyzed (Table 7). Only 1 in 33 patients with a diverting stoma developed local recurrence, and 5 developed distant recurrence. No difference in the recurrence rate was detected between groups. Distant relapse-free survival (D-RFS) and loco-regional relapse-free survival (L-RFS) was not different between the two groups (Fig. 1).

**Table 7:** Incidence of Distant Recurrence and Local Reccurrence.

Comparison of Recurrence in Patients with or without Diverting Stoma (N = 230)			
	Patients with (N = 34) (%)	Patients without (N = 196) (%)	*P*
Distant Recurrence (%)	5 (14.7)	27 (13.8)	NS
Local Recurrence (%)	1 (2.3)	5 (2.6)	NS

A two-tailed p value ≤ 0.05 was considered statistically significant; NS, not significant.

## Discussion

The purpose of this study was to investigate other benefits of temporary diverting stoma, except for the possibility in decreasing the incidence. The principal finding was its positive impact on recovery of AL after anterior resection of rectal tumors. In the non-sever group, it did reduce the mean recovery time, without influencing the length ofhospital days or economic burden, especially in patients who did not receive neo-CRT. However, we did not detect a positive impact on occurrence rate of severe AL, recovery time and its correlation with distant or local recurrence rate or relapse-free survival.

The purpose of temporary diverting stoma is to decrease pressure and feces contamination of the anastomosis. Adiverting stoma during the primary operation could reduce the occurrence of AL^10–13^. The Sweden randomized controlled trial compared 234 patients undergoing low anterior resection, randomized to the stoma (*n* = 116) and no stoma (*n* = 118) groups, and found a significantly lower rate of AL (10.3% vs. 28.0%), as well as lower rates of urgent re-operation,with stoma use. However, it did not influence the rate of late leakage (greater than 30 days postoperatively)^14^. On the contrary, one newly published study using propensity score matching to eliminate potential bias did not yield the same result^15^. They did find that a diverting stoma could possibly reduce the rate of re-laparotomy with symptomatic AL (grade C), which was also confirmed by other researchers^13,16^. We did not find a decreased rate of severe AL (grade C) after diverting stoma, mainly owing to the limited number of patients. Further studies with large cohorts are necessary. Moreover, our study did not include patients with grade A AL because of its difficulties in diagnosis. So the possible advantage of temporary diverting stoma might be underestimated.

We believe that even when AL does occur, a positive effect of temporary diverting stoma exists in terms of healing of the anastomosis. Interestingly, we did not identify the same efficacy in patients after neo-CRT. Preoperativeradiotherapy is widely applied to improve local recurrence or disease-free survival^17^.The functions of preoperative chemotherapy and radiotherapy on rectal cancer are different, with radiotherapy targeted to local control of tumors^18^. However, several large retrospective studies^19,20^ and randomized controlled trials^17,21^ have suggested that preoperative radiotherapy leads to AL. Although we could not evaluate the specific neoadjuvant treatment strategies of our patients in detail owing to data limitations, we believe that preoperative radiotherapy predominantly affects healing of the anastomosis; thus, radiotherapy overcame the positive influence of a diverting stoma, resulting in no improvement in recovery time among patients who underwent neo-CRT. In severe AL group, however, no difference was detected. Notably, patients with severe AL were always combined with other emergencies, like shock or sepsis, and evaluation of AL healing remained inaccessible due to the complicated individual treatment strategy. Moreover, possible benefit from diverting stoma could also be replaced by re-operation or intensive medical care.

We also investigated the length of hospital stay and costs after placement of a diverting stoma in non-severe group. In spite of secondary hospitalization and re-operation for closure, time and economic cost did not increase with stoma placement. Considering our results and previously published recommendations of a diverting stoma to decrease AL, along with the fact that AL did increase total clinical and economic burden^22^, the use of a diverting stoma might be a good choice.

It is commonly accepted that AL has a negative prognostic impact on local recurrence after restorative resection of rectal cancer^23^. Warrier *et al*. demonstrated a correlation of AL with local recurrence (OR = 1.61, 95% CI: 1.25–2.09; *p* < 0.001), but not distant recurrence (OR = 1.07, 95% CI: 0.87–1.33; *p* = 0.52)^24^. Considering this, we would like to investigate whether differences in recovery time correlated with local recurrence.We did not detect any trends related to L- and D-RFS. Only one patient developed local recurrence in the diverting stoma group. Due to the limited number of cases in our study cohort, it remains unclear whether a diverting stoma and shorter recovery time is associated with better disease-free survival.

The current study has several limitations that are common in retrospective investigations. One problem was the obvious bias in our study. Although we have a comparatively large numbers of patients with AL, groups based on various interventions are limited and unbalanced. We were also confined by the various length of follow-up time, as almost one-third of patients were followed for less than 2 years, leading to a low rate of recurrence.

Although we detected another benefit from temporary diverting stoma, we agree that temporary diverting stoma should be applied to specific groups of patients, not all of them. The duration of stoma, the possibility of closure, and patient quality of life should be further investigated. Further studies with a larger number of cases and longer follow-up period are required to confirm our findings, and to clarify the exact influence of a diverting stoma and AL on survival.

## Methods

### Patient selection and treatment

We retrospectively reviewed a database of patients treated in the Department of Colorectal Surgery at the Shanghai Cancer Center from June 2006 to December 2015. Patients who met the following criteria were selected: 20–80 years of age and preoperative performance status (PS) of 0–2. The exclusion criteria were previous intestinal resection or previous pelvic surgery. Patients with rectovaginal fistula were also excluded because we found a gigantic difference of incidence and recovery process, compared to those with simple AL. This would be investigated further.

Altogether 5035 patients underwent anterior resection of rectal tumor were studied, in which 323 patients conformed to the criteria. In our sub-group analysis, all patients developed AL after the primary operation. Clinical records, including personal histories, review of systems, pathologic diagnoses, and treatment strategies were available and complete.

All patients had a closed system drain inserted into the presacral space routinely. Generally, AL was defined as a defect of the intestinal wall at the anastomosis between the colon and rectum. Clinical signs and symptoms included fever and chills, detection of pus or feces from the drain or wound, and increased inflammatory markers, like leukocyte or neutrophil count, C-reaction protein level, or procalcitonin level. Rectoscopy, computed tomography and water-soluble contrast enema were also used to confirm the diagnosis, as well as to exclude simple pelvic abscess. Few patients developed AL after removal of drainage tubes, and the diagnosis was mainly based on the clinical symptom and image examination. After diagnosis of AL, conservative treatment, primarily drainage at full flush, were initially applied with or without intravenous antibiotics, based primarily on the treating clinicians’ preference.

This study was conducted according to the principles expressed in the Declaration of Helsinki and was approved by the Research Ethics Committee of the Fudan University Shanghai Cancer Center in China. All patients provided written informed consent.

### Outcome parameters and definitions

The following parameters were evaluated: sex, age, personal history including excessive smoking (>40 pack-years), long-term alcohol history, and body mass index (BMI), and preoperative diagnoses including diabetes, vascular disease, and anemia. Weight loss, American Society of Anesthesiologists (ASA) score, and neoadjuvant treatment (Neo-CRT) were also evaluated. Operative parameters included urgent surgery because of complete or incomplete intestinal obstruction, blood transfusion, and temporary diverting stoma, including distal ileum ostomy or transverse colonic ostomy, as well as distance to the anal verge. Pathological parameters, including maximum tumor size, TNM stage, lymphovascular invasion, perineural invasion, and extranodal tumor deposits also were assessed. AL recovery was defined as symptom alleviation, normalization of inflammatory indices, and clean drainage from the wound. After withdrawing of the drainage tube, AL was resolved without recurrence in all patients. In patients with temporary diverting stoma, length of hospital stay and cost were calculated including secondary hospitalization for stoma closure. All patients did not discharge from hospital until the medical proofs on AL, including symptoms and medical examinations, were completely cured, according to Chinese medical service system.

Severe AL was combined with fatal abdominal infection or peritonitis, requiring emergency re-operation. Non-severe AL, however, was not fatal and could be mostly cured by conservative treatment. Occurrence and recovery time were further investigated. Occurrence time of AL was defined as the duration between the primary operation and AL occurrence, while recovery time was defined as the length of hospitalization (LOH) duration between AL occurrence and recovery.

Distant relapse-free survival (D-RFS) was defined as the duration from primary tumor resection to distant metastasis, while loco-regional relapse-free survival (L-RFS) was defined as histologic or radiologic evidence of recurrent disease localized to the anastomosis. Patients with stage IV disease (*n* = 18) were excluded from the survival analysis. Altogether, 230 patients with complete follow-up data were included.

### Statistical analysis

All statistical analyses were performed using SPSS version 20.0 (IBM Corporation, Armonk, NY, USA). Chi-square tests or Fisher’s exact test for categorical variables was used to compare the application of a diverting stoma among groups. The independent t-test was applied for continuous and normally distributed variables. Frequencies were also compared via the Mann-Whitney U test for unpaired observations. D-RFS and L-RFS curves were calculated using the Kaplan-Meier analysis and compared using the log-rank test. Two-sided 95% CIs were calculated and all reported *p*-values are two-sided.

## Electronic supplementary material

Supplementary Table S1
