# Supplementary material for: Temporary Diverting Stoma Improves Recovery of Anastomotic Leakage after Anterior Resection for Rectal Cancer
Source: Sci Rep. 2017 Nov 21;7:15930. doi: 10.1038/s41598-017-16311-7 (PMC5698498; doi:10.1038/s41598-017-16311-7)
Supplement: Supplementary file 1 — Supplementary Table S1 [file 41598_2017_16311_MOESM1_ESM.doc]

**Temporary Diverting Stoma Improves Recovery of Anastomotic Leakage after Anterior Resection for Rectal Cancer**

Yuchen Wu, MD.^1,2^*, Hongtu Zheng, MD^1,2^*, Tianan Guo, MD.^1,2^, Adili Keranmu, MD^1,2^, Fangqi Liu, MD^1,2^, Ye Xu, MD^1,2^

^1^Department of Colorectal Surgery, Fudan University Shanghai Cancer Center, No. 270, Dong An Road, Shanghai 200032, China

^2^ Department of Oncology, Shanghai Medical College, Fudan University, No. 130, Dong An Road, Shanghai 200032, China

**Corresponding Authors:**

Corresponding author: Dr. Ye Xu, Department of Colorectal Surgery, Fudan University Shanghai Cancer Center, Shanghai 200032, China; Department of Oncology, Shanghai Medical College, Fudan University, 270 Dong’ an Road, Shanghai 200032, China. Phone number: 86-21-64175590-81108 Fax number: 86-21-64035387

Ye Xu email: xu_shirley021@163.com/ [xuye021@163.com](mailto:xuye021@163.com)

**First Author:**

Yuchen Wu

Email: antonor123@163.com

Hongtu Zheng

Email: zht5863@126.com

**Contributing Authors:**

Tianan Guo

Email: gta89757@126.com

Adili Keranmu

Email: adili117@163.com

Fangqi Liu

Email: 1151231949@qq.com

**Yuchen Wu and Hongtu Zheng contributed equally to this work and should be regarded as joint first authors.**

**Supplementary Table S1** Time Evaluation with Other Variables

| **Comparison of Time with Other Variables (Non-Severe Group, N=285)** | | | | | |
| --- | --- | --- | --- | --- | --- |
|  |  | **Occurrence Time**  **mean±SD, d** |  | **LOH**  **mean±SD, d** | ***P*** |
| Age | ≤58 | 6.8 ± 3.3 | NS | 20.0 ± 12.0 | NS |
|  | >58 | 6.9 ± 2.7 |  | 20.3 ± 12.0 |  |
| Sex | Male | 6.6 ± 3.1 | **0.042** | 19.6 ± 12.3 | NS |
|  | Female | 7.5 ± 3.3 |  | 21.9 ± 9.9 |  |
| Excess Smoking | YES | 6.8 ± 3.2 | NS | 19.4 ± 10.4 | NS |
|  | NO | 6.6 ± 3.1 |  | 20.3 ± 12.5 |  |
| Excess Alcohol | YES | 7.3 ± 3.3 | NS | 19.9 ± 10.9 | NS |
|  | NO | 6.6 ± 3.1 |  | 20.1 ± 12.2 |  |
| BMI | <20 | 6.2 ± 2.9 | NS | 17.8 ± 9.8 | NS |
|  | 20-24 | 6.5 ± 2.7 |  | 19.8 ± 11.4 |  |
|  | >24 | 7.1 ± 3.8 |  | 20.1 ± 13.4 |  |
| Vascular disease | YES | 6.7 ± 2.8 | NS | 19.4 ± 9.6 | NS |
|  | NO | 6.7 ± 3.2 |  | 20.3 ± 12.6 |  |
| Diabetes | YES | 6.7 ± 3.0 | NS | 18.6 ± 10.9 | NS |
|  | NO | 6.6 ± 3.7 |  | 20.3 ± 12.1 |  |
| ASA Score | 1-2 | 6.7 ± 2.6 | NS | 17.8 ± 10.1 | NS |
|  | 3-5 | 6.2 ± 2.4 |  | 18.4 ± 11.6 |  |
| Weight Lost ≥5kg | YES | 6.6 ± 4.2 | NS | 22.0 ± 11.0 | NS |
|  | NO | 6.7 ± 3.0 |  | 19.9 ± 12.0 |  |
| Anemia | YES | 5.9 ± 4.0 | NS | 22.8 ± 11.4 | NS |
|  | NO | 6.8 ± 3.0 |  | 19.7 ± 12.0 |  |
| Neo-CRT | YES | 7.0 ± 2.3 |  |  |  |
|  | NO | 6.9 ± 2.9 |  |  |  |
| Diverting Stoma | YES | 6.8 ± 3.2 | NS |  |  |
|  | NO | 6.6 ± 2.9 |  |  |  |
| Type of Surgery: Urgent | YES | 6.8 ± 3.3 | NS | 17.5 ± 7.2 | NS |
|  | NO | 6.7 ± 3.1 |  | 20.2 ± 12.2 |  |
| Blood Transfusion | YES | 3.5 ± 0.7 | NS | 22.5 ± 9.2 | NS |
|  | NO | 6.7 ± 3.1 |  | 20.0 ± 12.0 |  |
| Distance to anal verge | ≤5cm | 6.7 ± 2.9 | NS | 20.0 ± 12.2 | NS |
|  | >5cm | 6.8 ± 4.6 |  | 20.9 ± 9.8 |  |
| Drainage Flush Fluid with intravenous antibiotics |  |  |  | 17.9 ± 10.1 | NS |
|  |  |  |  | 19.8 ± 12.6 |  |
| Drainage Flush Fluid with Disinfectant |  |  |  | 18.9 ± 12.8 | NS |
|  |  |  |  | 20.9 ± 11.1 |  |
